# The psychosocial effects of being quarantined following exposure to COVID-19: A qualitative study of Lebanese health care workers

**DOI:** 10.1177/0020764020932202

**Published:** 2020-06-03

**Authors:** Mirna Fawaz, Ali Samaha

**Affiliations:** 1 Nursing Department, Faculty of Health Sciences, Beirut Arab University, Beirut, Lebanon; 2 Faculty of Public Health, Lebanese University, Zahle, Lebanon; 3 Faculty of Letters and Human Sciences, Lebanese University, Zahle, Lebanon; 4 Department of Biomedical Sciences, Lebanese International University, Beirut, Lebanon

**Keywords:** Psychosocial effects, quarantined, COVID-19, health care workers

## Abstract

**Background:**

Since the outbreak of the novel Coronavirus (COVID-19), health care professionals in Lebanon have been diligently serving as the frontline of defense. In the light of challenging economic and political circumstances, putting their community wellbeing as a priority, and abiding by quarantine and strict infection control measures, health care professionals risk both their physical and mental wellbeing.

**Objective:**

The aim of this study is to explore the psychosocial effects of being quarantined following exposure to COVID-19 among Lebanese health care professionals.

**Method:**

An exploratory qualitative research design was employed, where semi-structured interviews were carried out involving a sample of 13 Lebanese health care providers working at various COVID-19 units.

**Results:**

The qualitative analysis has revealed four themes namely ‘Fears of contracting and spreading the virus’, ‘Conflict between professional duty and family obligation’, ‘Stigma of being infected’, and ‘Inadequate or inaccurate information’.

**Conclusion:**

COVID-19 quarantine has been posing intense psychological challenges among Lebanese health care workers which are worsened at times by the economic instability; thus, health care policymakers are urged to take proper action nationwide to alleviate longlisting implications and support the health care providers in fulfilling their mission.

## Introduction

Upon the outbreak of the COVID-19 virus in Wuhan, China, which has spread to diverse communities and afflicted large numbers of people, the United Nations (UN) health agency has declared the virus as an epidemic. However, on 11 March 2020 and after the virus has made its way to infect even larger numbers in countries around the globe, such as Italy, Iran, and Spain, which have the highest numbers of cases and fatality rates, the World Health Organization (WHO) has officially declared the communication of COVID-19 as a pandemic, which means that it has the characteristics to qualify as an international phenomenon. This press conference raised the voice regarding the ‘alarming levels of the coronavirus spread, severity and inaction’, where WHO officials stated that they’re expecting numbers of cases and mortalities to further increase (Bogoch et al., 2020).

The initial case of coronavirus Lebanon was recorded on 21 February 2020 which after 20 days the government have decided to enforce a partial embargo, closed public and private institutions, including the airport where flights from and to Italy, South Korea, Iran, and China were banned, and Lebanese citizens abroad were given a duration of 4 days to return before the travel ban becomes effective (Tuite et al., 2020). To date, the country has recorded 333 cases and 5 deaths, where those numbers are actually on 15% of the actual cases; thus, the numbers amount to more than the ones officially documented, where each person can infect two or three people on average (WHO, 2020).

In light of the pressing economic crisis that has been intensifying constantly and impeding Lebanon’s ability to properly respond to this health crisis, the outbreak has triggered a state of panic among the public. The spread of COVID-19 has taken the health care system off guard in Lebanon, especially that the hospitals are not properly equipped to receive large numbers of cases and it is not doable to dedicate full hospitals to the treatment of the COVID-19 cases as Lebanese hospitals whether governmental or private take on around 8,000 patients on a daily basis, some of which are serious cases. Lebanon also has about 750 functional ventilators, only 250 of which have been dedicated to coronavirus cases, which adds to the pressure of the issue (Ministry of Public Health [MOPH], 2020).

Health professionals have become the most vulnerable population to contract the virus and represented over 20% of those who actually contracted the disease. Contracting COVID-19 became a major worry for health care staff with the rising number of COVID-19-infected personnel; in that regard, the order of nurses in Lebanon has indicated in a recent report which showed that at least 15 nurses have caught the infection. This has contributed to the skepticism of insufficient preventive measures (MOPH, 2020). The pandemic has been posing a huge responsibility and a heavy psychological hurdle to stay safe and keep their loved ones safe as well while still fulfilling their duties. Conflicting accounts of health care professionals’ readiness to carry out high-risk tasks (Maunder et al., 2003; Schull & Redelmeier, 2003) prompted revived controversy about whether health care personnel had a right to decline to operate in high-risk conditions. Most workers feel torn in their positions as health care professionals and parents, they feel a moral obligation, but they still expressed anxiety and worry for their children being afflicted with the virus because of them. The nurses exposed to the virus have placed themselves in quarantine in order to prevent further outbreak especially to their families; thus, they have started renting out flats and apartments next to the hospitals which they are barely even affording.

Similar challenges that have arisen due to COVID-19 outbreak have led to various psychological and emotional disorders and illnesses among many health care professionals in China, United States, Canada, Taiwan and Hong Kong who were caring for patients with the disease (Chen et al., 2020; Duan & Zhu, 2020). The psychiatric illness most frequently correlated with disaster-related events in the previous research is post-traumatic stress disorder (PTSD), but reports have also shown that comorbid depression is prevalent in people after a disaster (Geng et al., 2019; Zhen et al., 2019).

Lebanese health care professionals are faced with a demanding reality that is might also give rise to psychological symptomatology in the long run. Therefore, a qualitative evaluation associated with the repercussions of the risk of exposure to the virus may reveal a variety of perspectives, where the psychological and emotional viewpoints of them might be more significantly correlated to consequential mental afflictions than the objective aspects. Nonetheless, past COVID-19 disease research did not analyze the relationship between contextual expectations of outbreak-related danger and corresponding rates of mood disorders. In fact, little has been understood regarding the causes that could be correlated with the post-disaster prevalent depression. Although it has been seen that philanthropic motivation to aid is beneficial against psychological distress (Navarro-Mateu et al., 2019), the paucity of research that examined its impact on post-disaster depression have provided conflicting results. Thus, the aim of the present study is to examine the psychosocial effects of being quarantined following exposure to COVID-19 among Lebanese health care workers.

## Methods

The study has adopted an exploratory qualitative research design to obtain a profound understanding of the psychosocial experiences of quarantined nurses. The sample included 13 participants acting in the COVID-19 teams at Lebanese hospitals. The study employed a convenient sampling technique due to the limited number of available, accessible, and eligible candidates to be included in this study. The eligibility criterion was set to be health care professionals who were quarantined due to the occupational exposure to COVID-19 cases, where they had to take measures such as leaving their families, renting out apartments or staying in separate specialized hospital dormitories to prevent a possible spread of the virus in case they have contracted it. An email was sent to the possible participants, where the aim of the study was explained and they were asked to provide written informed consent. Institutional Review Board approval was procured as the study has abided by the ethical guidelines of research (IRB number: ECO-R-15). The participants took part in hour-long semi-structured interviews, were due to the strict schedules, long working hours, and the need for precautions, 11 interviews were done through telephone while 2 were done face-to-face. The researchers have kept carrying out the interviews until data saturation was reached. The researchers have agreed with the subjects at a convenient time for the interview so that they would be able to freely express genuine opinions. The researchers took alternating turns in moderating the interviews in order to prevent ‘moderator dominance’. The researchers thus posed the following questions: ‘How do you describe living through the quarantine experience?’, ‘How is quarantine affecting you?’, ‘How do you perceive your situation regarding contracting and spreading COVID-19?’ and ‘How is COVID-19 affecting your work as a healthcare professional?’. A phone call recorder application was used to keep a record of the interviews, while a regular recorder application was used to tape the face-to-face interviews. Qualitative narratives were translated into the English language and have been examined by employing an inductive thematic content analysis, which involved reading verbatim, open text labeling, establishing classes and abstracting final themes (Burnard et al., 2008; Elo & Kyngäs, 2008; Hays & Singh, 2011). The analysis was carried out separately by each researcher, and then they discussed their findings until a consensus was reached, while not involving their own perception during that process. The researchers read them verbatim provided by the respondents multiple times in order to get a genuine understanding of the experience lived by the health care professionals. The phrases were given a reflective word that captures the essence of the datum provided and after that the words grouped and aggregated into themes, which were addressed by the two authors to make sure that they are representative of the studied experience. In order to enhance the trustworthiness of the study and avoid biases, multiple processes were undertaken by the researchers according to what has been described in past literature in the area of qualitative research (Krefting, 1991). Concurrent analysis ensured that emerging phenomena could be explored in simultaneous interviews to achieve a thorough interpretation of concepts. Both researchers had employed the same structure for the interviews and questions used. The authors discussed the concepts formed and did not ignore any aspect of the findings. Various verbatim excerpts were used to explain the research’s outcomes, and this provided the respondents with a voice in this research article. Additionally, coordination with qualified professionals was established where they used control procedures to check the findings. The analysis was conducted autonomously by two scholars. The writers addressed the topic of interpreting and evaluating observations that culminated in agreement on shared themes (Speziale et al., 2011). Besides peer checking, the investigators employed the member-checking approach to developing credibility and validation. After reviewing each discussion, the respondents were sent back the themes and descriptions to verify the findings. The investigators did, in addition, employ the external member-checking method to guarantee that the study results were transferable. To ensure generalizability through contexts, the results of the research were introduced to a variety of health care professionals who had not participated in the analysis and were requested to determine the similarity between the findings of the study and their own experiences.

## Results

### Characteristics of the respondents

The research sample comprised 13 health care professionals, 9 (69.2%) of which were nurses while 4 (30.8%) were physicians, where both were caring for COVID-19 cases and have been quarantined due to their exposure to active COVID-19 patients. The results showed that 11 (94.61%) of the participants were married while 2 (15.38 %) of them were single. In addition, 7 (53.8%) of the respondents had children while 6 (46.2%) did not have children, where 8 (61.5%) of them were obliged to rent out apartments near their respective hospitals while 5 (38.5%) of them were able to stay at hospital-provided dormitories.

### Phenomenology

The phenomenological data analysis has given rise to multiple themes that have been consistent among the recounts of the participants namely ‘Fears of contracting and spreading the virus’, ‘Conflict between professional duty and family obligation’, ‘Stigma of being infected’ and ‘Inadequate or inaccurate information’.

#### Fears of contracting and spreading the virus

The nurses and physicians have commonly expressed their concern regarding contracting the COVID-19 virus during their duty and spreading it to the community and most importantly to their families, which is making them follow infection control measures more obsessively and therefore taking quarantine actions. For instance, one nurse said,

Since the start of the outbreak, I have started to be really obsessed with handwashing and disinfecting everything I touch. After I started serving in the Corona unit, the fear got even worse as I am now more vulnerable to catch the virus and spread it to my family . . . that minute I knew I was going to say goodbye to my family for my safety and theirs. I don’t want to be the source of their illness. (N3)

Another nurse indicated,

Even though I take all the precautions needed while caring for a COVID-19 patient, there’s always this voice at the back of my mind saying that the virus might have seeped in somewhere . . . I feel like it’s always not enough whatever I am doing, it always feels like I might have contacted the virus and I might spread it to someone else. (N6)

A physician similarly proclaimed,

The first few days of serving in the unit I was doing fine but then I started anxiously checking my vitals . . . always desperately looking for any symptoms I’m showing that might indicated my affliction as if my mind is always playing tricks on me that I might be infected and I am not aware of it. (P1)

#### The Conflict between professional duty and family obligation

The decision to care for COVID-19 patients presented a dilemma among the interviewed health care providers and brought a sense of frustration between their duties toward family and obligation to their profession. For instance, one of the nurses said,

It was a heavy decision at first, to isolate myself from the world and most importantly my family. I was torn; I could be afflicted, I could even infect my loved ones. On the flip side, I always have been dedicated to my patients and didn’t want to stop now. It was really hard to tell my family . . . my husband . . . my kids . . . I was the one to take care of the household, cook, clean, take care of the children, and my husband’s needs . . . it took some time to reach a consensus but here I am. (N1)

Also, another nurse proclaimed,

Leaving my wife and children at home alone was hard for me, especially that I was the one to make sure everything is set for them before I leave to work. I am a diligent nurse, I stand with my code of ethics, however, it was the first time I was put in such a conflicting situation especially that it posed risk for both myself and my family, but they were supportive and I was more than ready to fulfill my duty. (N2)

A physician also shared by saying,

Being a doctor means that you have to deliver optimal care no matter what . . . in order to fulfill my obligation I had to talk to other family members to tend to my mother and father. When I told them I was going to be working with COVID-19 patients they freaked out especially that they were being constantly fed with information through the TV which they didn’t know how to receive . . . their reaction was expected, but they finally came around . . . I am doing my job, but sometimes I still get that feeling of guilt that I am not doing my duty towards them too . . . to keep them safe and tend to them. (P3)

#### Stigma of being infected

The participants reported that working in a hospital in the time of the outbreak gave rise to negative reactions from people at first, and when they knew they were working in the coronavirus unit, it was even more exaggerated. One nurse said,

Even before working in the COVID-19 unit, my friends and some family members refused to see me because I might be carrying the virus. When I volunteered, the situation got worse especially that I was quarantined. Everyone was perceiving me as infected. I don’t know how they would receive me after the outbreak . . . would they still have the same fear? (N5)

Another nurse said,

At the start of the outbreak, I didn’t even allow myself to get in contact with other people especially my old family members like grandparents; they are vulnerable and one never knows . . . but like at a certain point, my aunt was standing way far from me when I saw her in the street . . . she felt like I was infected . . . treated me as if I am the virus . . . it was weird. After working in the COVID-19 unit . . . man it was bad! I kept getting WhatsApp messages joking about me being infected with corona. (N3) (P2)

Another physician also had a similar experience,

Before quarantine, it was very hard to get a lift . . . no one accepted to take me to the hospital they were all concerned and thought that the area surrounding the hospital is infested and they would even catch the virus from the air surrounding the hospital. Now that I am quarantined for working in the COVID-19 unit . . . my friends and family do have an inclination to treat me as I am contagious . . . it brings frustration and sometimes even anger really. (P4)

#### Inadequate or inaccurate information

The final emergent theme which the participants expressed revolved around the fact that inaccurate information was circulating among the general public as well as among health care professionals. One nurse said,

We are always bombarded with information through WhatsApp and other social media outlets, and you don’t know which information is accurate and which is a rumor. We get news about huge numbers of cases getting transported to the units . . . other news about patients being carried on airplanes . . . we are always on guard and it works my nerves. (N7)

Another nurse proclaimed,

Some pieces of information are being withheld or at least that what we are hearing across media platforms . . . that the numbers given by health officials and government officials are not true . . . on the other hand rumors tend to break out that we are receiving hundreds of cases within hours and we get nervous as of how to deal with them but they turn out to be fake news . . . the news circulates even within the hospital itself. (N9)

Another nurse highlighted,

. . . certain official and needed measures were even subject to debate . . . have we closed borders or have we not? What are the measures we need to do by policy . . . what are we to expect? Can we go out or will we be fined . . . it was all blurry. (N1)

A physician also recounted, ‘The huge amount of fake news is confusing . . . sometimes we get overwhelmed preparing for incoming cases that turn out to be not true’ (P1).

## Discussion

In the light of any health emergency, health care providers are always at the forefront, putting up their lives for the health of their communities. This personnel especially the ones working in high-risk units are faced with tremendous psychological challenges, let alone the implications being associated potential with being infected. This was prevalent during the SARS and then the EBOLA outbreak (Lin et al., 2007; Lehmann et al., 2015); however, the COVID-19 differs in its geographic and pathogenetic nature which has infected higher numbers of people in diverse areas of the world and caused even further panic among the population. However, health care providers being at the center of the storm, having to risk their health and their families’ stability they are expected to deal with heavy psychological stress. The results of this study showed that upon working in the COVID-19 and being quarantined due to exposure, health care providers in Lebanon started showing symptoms such as severe stress, frustration, confliction, and anger, as a result of separation, stigmatization, fear of contracting the coronavirus, the clash between the obligation to family and profession, in addition to confusing information and health policy. Also, a recent study by Xu and Zhang (2020) had similar findings to this study where nurses were found to have negative emotional outcomes due to COVID-19 and quarantine such as anxiety, depression, and fear. Consistently, many nurses have displayed contradictory responsibilities as public care providers and parents throughout the SARS epidemic. However, they felt accountable, benevolent, and proficient. Yet, they were distressed and were accused of infecting their family members (Maunder et al., 2003). Another recent study also found that quarantined health professionals and population during COVID-19 quarantine might lead to anxiety and stress disorders due to fear of infection and spreading the infection to loved ones, frustration, inadequate supplies, and information, as well as stigmatization of health care providers as being contagious (Brooks et al., 2020). Liu et al. (2020) found out that the COVID-19 outbreak has exposed possible vulnerabilities in mental wellbeing programs during crises, while also measuring the effectiveness of health care personnel and health systems. Duan and Zhu (2020) reported the rise in psychiatric disorders including fear, depression, and tension during this outbreak. Similarly, research by Nickell (2004) found that nearly 20% of the community experienced mental distress after SARS, and the prevalence among nurses in Toronto was quite significant. Furthermore, our results were consistent with Khalid et al. (2016) which found that anxiety was prevalent among health care staff during the MERS-CoV epidemic, where the main sources of stress were fear of infection and transmitting disease to their families.

## Conclusion

The quarantined health care providers that we have interviewed have expressed a spectrum of feeling through their lived experience from fear to anger, confliction, frustration, and anxiety, they were first and foremost dedicated to their profession and to caring for their patients. The results of this study showed that health care personnel are being psychologically challenged through quarantine and need to have clear health communication from nursing managers, leaders, and policymakers. In addition, proper health communication should be provided to the public so that they can know the reality of the situation and bypass any misconceptions and stigmatization. Moreover, moral and financial support should be offered to this quarantined personnel from governmental and non-governmental health policymakers in order for them to be able to maintain their health and their families’ stability. Mental health caregivers should raise this issue to health leadership and media in order to support these professionals in these hard times.
